# Analytical model for managing hypotony after implantation surgery of a glaucoma drainage device

**DOI:** 10.1007/s10237-021-01494-w

**Published:** 2021-07-23

**Authors:** R. Agujetas, B. Kudiesh, J. I. Fernández-Vigo, Julián García-Feijóo, J. M. Montanero

**Affiliations:** 1 Depto. de Ingeniería Mecánica, Energética y de los Materiales and Instituto de Computación Científica Avanzada (ICCAEx), Universidad de Extremadura, E-06006 Badajoz, Spain; 2 Department of Ophthalmology, Puerta de Hierro University Hospital, Majadahonda, Madrid, Spain; 3 International Center of Advanced Ophthalmology, Madrid, Spain; 4 Department of Ophthalmology, San Carlos Clinical Hospital, Health Research Institute (IdISSC), Universidad Complutense. OFTARED, Madrid, Spain; 5 International Center of Advanced Ophthalmology, Madrid, Spain

**Keywords:** Aqueous humor flow, Intraocular pressure, Glaucom, Glaucoma drainage device

## Abstract

The main aim of glaucoma treatment is to reduce the intraocular pressure (IOP). One of the most common surgical treatments of glaucoma is the implantation of a glaucoma drainage device to drain the aqueous humor from the anterior chamber to a filtration bleb, where the aqueous humor is absorbed. In some cases, the excess of drainage causes ocular hypotony, which constitutes a sight-threatening complication. To prevent hypotony after this intervention, surgeons frequently introduce a suture into the device tube, which increases the hydraulic resistance of the tube and, therefore, the IOP. This study aims to provide an analytical model to correct hypotony following implantation surgery of a glaucoma drainage device, which may help glaucoma surgeons decide on hypotony treatment. The results indicate that the IOP after implanting a cylindrical tube around 300 μm in diameter is essentially the same as that built up in the filtering bleb and can hardly be controlled by introducing a straight suture unless the suture diameter is slightly lower than that of the tube. On the contrary, when the tube diameter is smaller than, for example, 100 μm, significant reductions of the IOP can be obtained by introducing a thin suture into the tube.

## Introduction

Glaucoma is one of the main causes of irreversible blindness worldwide. The increased intraocular pressure (IOP) is considered the most important and only changeable risk factor for its development (Congdon et al 2004). The IOP increase usually occurs due to the difficulty in the outflow of aqueous humor rather than due to increased aqueous humor production (Ethier et al 2004). The main objective of the glaucoma treatment is the reduction of IOP through pharmacological therapy, laser, traditional filtering surgery, or glaucoma drainage devices (Heijl and Traverso 2017). Glaucoma drainage devices are implanted when the IOP raises up to values in the range 25–45 mm Hg to drive the aqueous humor from the anterior chamber to a filtration bleb formed in the subconjunctival space, where the aqueous humor is absorbed, and so the IOP is regulated (Tseng et al 2017; Aref et al 2017).

**Fig. 1:**
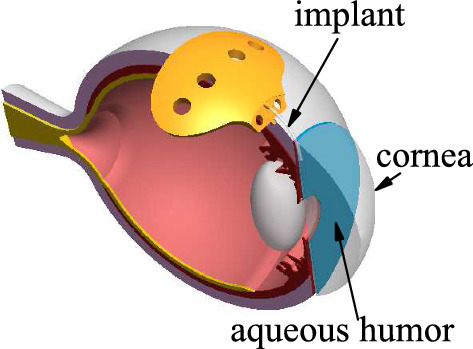
The Baerveldt implant (Advanced Medical Optics, Inc., Santa Ana, CA, USA)

One of the biggest challenges of the implantation surgery of a glaucoma drainage device is the avoidance of hypotony (IOP in the range 2–6 mm Hg), which may lead to sight-threatening complications including corneal edema and macular edema (Gedde et al 2012). Glaucoma drainage devices are divided into valved and non-valved implants depending on whether they include a flow restriction mechanism to avoid hypotony or not. The most commonly used valved device is the Ahmed glaucoma implant (New World Medical, Rancho Cucamonga, CA, USA), which allows only unidirectional flow from the anterior chamber to the subconjunctival space with a minimum opening pressure of around 5 mm Hg by using a Venturi implant mechanism (Coleman et al 1995). The Baerveldt implant (Advanced Medical Optics, Inc., Santa Ana, CA, USA) is the most used non-valved device (Fig. 1). To prevent hypotony, surgeons usually either do an external tube ligature by an external suture or an internal occlusion by introducing the suture into the device tube. This intervention allows the IOP to reach values in the range 8–12 mm Hg. Hypotony can also occur with Ahmed valved devices despite the valve mechanism. For this reason, glaucoma surgeons have proposed to introduce a suture inside the lumen of these implants to increase the IOP (Lim and Hwang 2018; Song and Hwang 2020; Pollmann et al 2020; Vergados et al 2019; Chen 2017; Mavrommatis et al 2019; Rietveld and van-der Veen 2004). Although this intervention may be a good surgical maneuver, there is no standard indication about which suture diameter should be used as a function of the IOP before and after the implant surgery and the diameter and length of the drainage device. In fact, the resulting IOP seems to be unpredictable and hardly reproducible under similar conditions (Rietveld and van-der Veen 2004). The present study aims to provide an analytical model to manage hypotony after implanting a glaucoma drainage device, which may help glaucoma surgeons decide on hypotony treatment.

The aqueous humor flow in the human eye has been studied on many occasions (Canning et al 2002; Fitt and Gonzalez 2006; Kapnisis et al 2009; Siggers and Ethier 2012) to examine different aspects of the problem, such as the formation of Krukenberg’s spindle (Heys and Barocas 2002), the effect of a laser iridotomy (Yamamoto et al 2006; Dvoriashyna et al 2017), the spontaneous reattachment of a previously detached Descemet membrane (Ismail et al 2013), the mechanical interaction between the aqueous humor and iris (Wang et al 2016), the fluid-dynamic effects of posterior-chamber and iris-fixed phakic intraocular lens (Kawamorita et al 2012; Repetto et al 2015; Tweedy et al 2017; Khongar et al 2017; Fernández-Vigo et al 2018; Agujetas et al 2019), the oscillatory and steady streaming flow in the anterior chamber of the moving eye (Abouali et al 2012; Dvoriashyna et al 2019), and the hypotensive efficacy and safety of minimally invasive glaucoma surgery devices (Kudsieh et al 2020). The information obtained from numerical simulations of the aqueous humor flow has proved useful in clinical practice.

The aqueous humor injected by the ciliary body flows radially across the posterior chamber toward the pupil and then pours into the anterior chamber through this orifice. When the eyelid is closed, the aqueous humor also moves radially in the anterior chamber toward the trabecular meshwork and finally goes through this tissue area draining into a set of tubes called Schlemm’s canal. This flow takes place at speeds lower than $10^{-3}$ mm/s with an essentially uniform reduced (piezometric) pressure until the aqueous humor reaches the trabecular meshwork. There, a pressure drop of the order of some mm Hg takes place due to the high hydraulic resistance offered by this tissue. When the eyelid is open, and the body is upright, natural convection drives the flow in the anterior chamber due to the difference between the body temperature and that of the posterior cornea. This motion takes place with velocities of the order of $0.1-1$ mm/s and with an associated pressure gradient of the order of $10^{-4}$ mm Hg. Natural convection does not affect the outflow across the meshwork due to the disparity between the pressure gradients associated with those flows.

As mentioned above, the outflow through the trabecular meshwork in a healthy eye is complemented with, or even entirely replaced by, a drainage device in an eye suffering from glaucoma. The pressure drop across the hydraulic system consisting of the drainage device and the filtration bleb is also much larger than that associated with natural convection, and therefore, this convection does not alter the drainage of aqueous humor from the anterior chamber to the subconjunctival tissue (Kudsieh et al 2020). This implies that the IOP resulting from the device implantation is independent of the location and orientation of the device in the anterior chamber. Besides, the smallness of the flow speed and length scales renders the flow dominated by viscosity, which greatly simplifies the problem. Thus, the calculation of the IOP following glaucoma surgery becomes analytically tractable. In this paper, we provide the analytical expressions which allow calculating the IOP after introducing a suture inside the glaucoma device as a function of the IOP before and after implanting the device, the device’s inner diameter and length, and the suture diameter.

## Analytical predictions

The IOP following the implantation of a glaucoma drainage device and the flow rate drained by the implant can be calculated analytically. The Reynolds number characterizing the flow in the implant takes very small values, the flow fully develops right at the entrance, and, therefore, the pressure drop across the device obeys to the generalized Hagen-Poisuille formula

1 $$\begin{aligned} p_\mathrm{c}-p_\mathrm{b}=\frac{128\, {{\mathscr {S}}} L_\mathrm{v} \mu Q_i}{\pi D_\mathrm{h}^4}, \end{aligned}$$

where $p_\mathrm{c}$ and $p_\mathrm{b}$ are the IOP following glaucoma surgery and in the filtering bleb, respectively, ${{\mathscr {S}}}$ is a dimensionless constant which depends on the shape of the implant cross-section, $D_\mathrm{h}$ and $L_\mathrm{v}$ are the implant hydraulic diameter and total length, respectively, $\mu$ the aqueous humor viscosity, and $Q_i$ the flow rate evacuated by the implant. The shape factor ${{\mathscr {S}}}$ of an implant with arbitrary shape can be calculated by numerically solving the dimensionless Poisson equation $\nabla ^2\hat{v}^2=1$ with $\hat{v}=0$ at the contour of the implant scaled such that $D_\mathrm{s}=1$. Then,

2 $$\begin{aligned} {{\mathscr {S}}}=\frac{\pi }{128}\left( \int _S \hat{v}\, \mathrm{d}S\right) ^{-1}, \end{aligned}$$

where *S* is the cross-sectional area of the scaled implant. If the implant is a cylindrical tube of diameter $D_i$, then $D_\mathrm{h}=D_i$ and ${{\mathscr {S}}}=1$. If we introduce a cylindrical suture of diameter $D_\mathrm{s}$ into the tube, then $D_\mathrm{h}=D_i-D_\mathrm{s}$. If the suture is placed coaxially with the tube, then

3 $$\begin{aligned} {{\mathscr {S}}}=\left( 1-\frac{D_\mathrm{s}}{D_i}\right) ^{4}\left\{ 1-\left( \frac{D_\mathrm{s}}{D_i}\right) ^4+\frac{[1-(D_\mathrm{s}/D_i)^2]^2}{\ln (D_\mathrm{s}/D_i)}\right\} ^{-1}. \end{aligned}$$

**Fig. 2:**
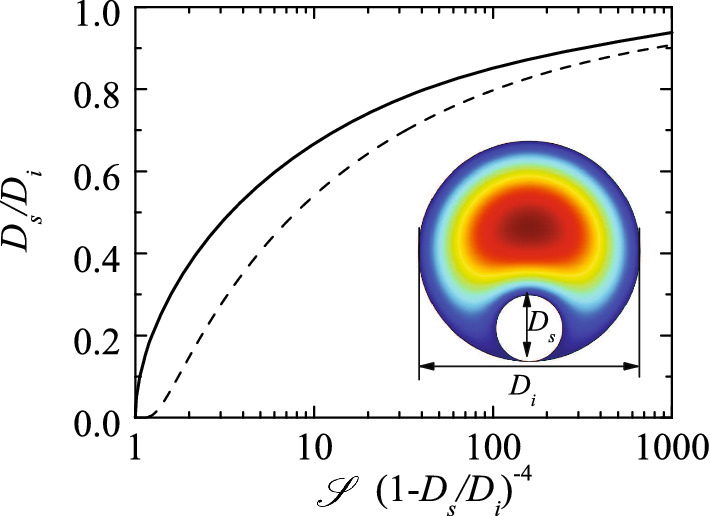
The ratio $D_\mathrm{s}/D_i$ as a function of the shape factor ${{\mathscr {S}}}$ when a cylindrical suture is introduced concentrically (dashed line) (Eq. (3)) and when it rests on the inner wall of the implant (solid line). The inset shows the isolines of the velocity magnitude in the latter case

If the suture rests on the inner implant wall, then ${{\mathscr {S}}}$ has to be calculated numerically. Figure 2 shows the relationship between ${{\mathscr {S}}}$ and $D_\mathrm{s}/D_i$ in the two cases mentioned above. The shape factor ${{\mathscr {S}}}(D_\mathrm{s}/D_i)$ in the non-axisymmetric case takes smaller values than in the axisymmetric one, which implies that the hydraulic resistance is larger in the latter case. Numerical simulations, including the effect of natural convection, have shown the validity of (1) for calculating the pressure drop in a glaucoma device without the suture implanted in the anterior chamber (Kudsieh et al 2020).

The trabecular meshwork and Schlemm’s canal offer a hydraulic resistance to the aqueous humor flow jointly. Because of the smallness of the Reynolds number in this region, that resistance does not depend on the flow rate and can be calculated as the ratio of the pressure drop across this system before surgery to the flow rate $Q_0=1.7$ μl/min evacuated by it. This approximation holds unless significant tissue deformation occurs. It has been hypothesized that the collapse of Schlemm’s canal can contribute to the hydraulic resistance of the system. The flow rate $Q_0$ mentioned above is calculated as that injected by the ciliary body, $Q_{\mathrm{cb}}=2$ μl/min, minus the one removed by the uveoscleral pathway, $Q_{\mathrm{up}}=0.15 Q_{\mathrm{cb}}$. The dependency of these two flow rates on the IOP can be neglected (Gardiner et al 2010).

Following the implantation of a glaucoma drainage device, the aqueous humor moves out of the bleb, gets into the surrounding sub-conjunctival tissue, and is absorbed by the sub-conjunctival capillaries. Both the sclera and conjunctiva are assumed to be impermeable, and, therefore, they act as fluid barriers. Gardiner et al (2010) described in detail the transport and absorption of aqueous humor taking place in the sub-conjunctival tissue. As explained in Appendix, the flow rate evacuated by the implant $Q_i$ under steady conditions verifies the equation

4 $$\begin{aligned} (p_\mathrm{b}-p_\mathrm{r})=R_\mathrm{b} Q_i, \end{aligned}$$

where $p_\mathrm{r}$ is a (constant) pressure of reference and $R_\mathrm{b}$ is a property of the sub-conjunctival tissue, which plays the role of an effective hydraulic resistance. According to the estimates of Gardiner et al (2010), $p_\mathrm{r}\simeq 0$.

If the IOP following glaucoma surgery, $p_\mathrm{c}$, is greater than the Schlemm’s canal (the episcleral venous) pressure, $p_{\mathrm{ev}}$, then the aquous humor can flow across the trabecular meshwork. Then, the IOP following glaucoma surgery is (Kudsieh et al 2020)

5 $$\begin{aligned} p_\mathrm{c}=\frac{\pi D_h^4 p_\mathrm{b}(p_\mathrm{g}-p_{\mathrm{ev}})+128\, {{\mathscr {S}}} L_\mathrm{v} p_\mathrm{g} Q_0 \mu }{\pi D_h^4 (p_\mathrm{g}-p_{\mathrm{ev}})+128\, {{\mathscr {S}}} L_\mathrm{v} Q_0 \mu }, \end{aligned}$$

where $p_\mathrm{g}$ is the IOP before surgery. The flow rate filtered by the implant is

6 $$\begin{aligned} Q_i=\frac{\pi D_h^4 Q_0 (p_\mathrm{g}-p_\mathrm{b})}{\pi D_\mathrm{h}^4 (p_\mathrm{g}-p_{\mathrm{ev}})+128\, {{\mathscr {S}}} L_\mathrm{v} Q_0 \mu }, \end{aligned}$$

and coincides with that at which the aqueous humor filters from the bleb. The drop of pressure across the implant can be calculated from Eq. (6) and the Hasen-Poiseuille formula (1):

7 $$\begin{aligned} p_\mathrm{c}-p_\mathrm{b}=\frac{128\, {{\mathscr {S}}} L_\mathrm{v}(p_\mathrm{g}-p_\mathrm{b}) \mu Q_0 }{\pi D_\mathrm{h}^4 (p_\mathrm{g}-p_{\mathrm{ev}})+128\, {{\mathscr {S}}} L_\mathrm{v} Q_0 \mu }. \end{aligned}$$

Equation (5) can be used to calculate the implant hydraulic diameter $D_\mathrm{h}$ and length $L_\mathrm{v}$ as a function of the target pressure $p_\mathrm{c}$:

8 $$\begin{aligned}&D_\mathrm{h}=\left[ \frac{128\, {{\mathscr {S}}} L_\mathrm{v} Q_0 \mu (p_\mathrm{g}-p_\mathrm{c})}{\pi (p_\mathrm{c}-p_\mathrm{b})(p_\mathrm{g}-p_{\mathrm{ev}})}\right] ^{1/4}, \end{aligned}$$

9 $$\begin{aligned}&L_\mathrm{v}=\frac{\pi D_\mathrm{h}^4(p_\mathrm{g}-p_{\mathrm{ev}})(p_\mathrm{c}-p_\mathrm{b})}{128\, {{\mathscr {S}}} Q_0\mu (p_\mathrm{g}-p_\mathrm{c})}. \end{aligned}$$

If the IOP following glaucoma surgery, $p_\mathrm{c}$, is lower than the Schlemm’s canal (the episcleral venous) pressure, $p_{\mathrm{ev}}$, then the aqueous humor cannot flow across the trabecular meshwork. Then, the flow rate filtered by the implant is $Q_0$ (that injected by the ciliary body minus the one removed by the uveoscleral pathway), and the IOP following glaucoma surgery is

10 $$\begin{aligned} p_\mathrm{c}=p_\mathrm{b}+\frac{128\, {{\mathscr {S}}} L_\mathrm{v} \mu Q_0}{\pi D_\mathrm{h}^4}. \end{aligned}$$

The drop of pressure across the implant can be calculated from Eq. (10). Equation (10) can be used to calculate the implant hydraulic diameter $D_\mathrm{h}$ and length $L_\mathrm{v}$ as a function of the target pressure $p_\mathrm{c}$:

11 $$\begin{aligned}&D_\mathrm{h}=\left[ \frac{128\, {{\mathscr {S}}} L_\mathrm{v} Q_0 \mu }{\pi (p_\mathrm{c}-p_\mathrm{b})}\right] ^{1/4}, \end{aligned}$$

12 $$\begin{aligned}&L_\mathrm{v}=\frac{\pi D_\mathrm{h}^4(p_\mathrm{c}-p_\mathrm{b})}{128\, {{\mathscr {S}}} Q_0\mu }. \end{aligned}$$

The above expressions involve the bleb pressure $p_\mathrm{b}$, which is generally unknown in clinical practice. As will be shown in the next section, this parameter does not intervene in the correction of hypotony following glaucoma surgery.

## Correction of hypotony following glaucoma surgery

Suppose that the IOP after implanting a glaucoma drainage cylindrical device, $p_\mathrm{c}^{(1)}$, is significantly lower than the desired one. To correct this hypotony, the surgeon introduces a suture of diameter $D_\mathrm{s}$ into the implant of diameter $D_i$, which modifies the value of both the shape factor ${{\mathscr {S}}}$ and the hydraulic diameter $D_\mathrm{h}=D_i-D_\mathrm{s}$ in Eq. (1). Consequently, the hydraulic resistance across the implant increases, and so does the IOP. The shape factor ${{\mathscr {S}}}_2$ after this correction can be calculated in terms of the ratio $D_\mathrm{s}/D_i$ (Fig. 2). The suture density is different from that of the aqueous humor. Neglecting the bending stiffness of the suture and the possibility that it bends, one can suppose that it moves inside the implant until it touches the wall tube. For completeness, we will also consider the case in which the suture remains at the center of the tube.

**Fig. 3:**
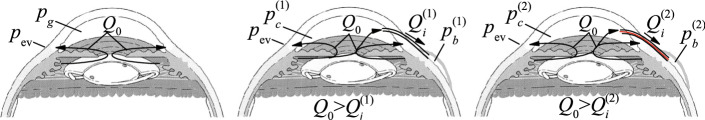
Sketch showing the flow of aqueous humor before surgery (left), after the implanting the drainage device (center), and after introducing a suture into the implant (right). The aqueous humor circulation corresponds to the case in which $p_\mathrm{c}^{(1)},p_\mathrm{c}^{(2)}>p_{\mathrm{ev}}$, and, therefore, the aqueous humor flows across the trabecular meshwork after the first and second interventions ($Q_0>Q_i^{(1)},Q_i^{(2)}$). The sketch has been adapted from Fig. 1 of Moon et al (2007)

We can make use of the above formulae to calculate the value of $D_\mathrm{s}$ leading to the prescribed value $p_\mathrm{c}^{(2)}$ of the IOP. Assume that the morphology of the bleb does not change after introducing the suture, and, therefore, nor does the bleb effective hydraulic resistance $R_\mathrm{b}$. In this case,

13 $$\begin{aligned} R_\mathrm{b}=\frac{p_\mathrm{b}^{(1)}-p_\mathrm{r}}{Q_i^{(1)}}=\frac{p_\mathrm{b}^{(2)}-p_\mathrm{r}}{Q_i^{(2)}}, \end{aligned}$$

where $p_\mathrm{b}^{(1)}$ and $p_\mathrm{b}^{(2)}$ are the bleb pressures following the first and second interventions, respectively, and $Q_i^{(1)}$ and $Q_i^{(2)}$ are the corresponding flow rates at which the aqueous humor filters across the belb (Fig. 3).

The case $p_\mathrm{c}^{(1)},p_\mathrm{c}^{(2)}>p_{\mathrm{ev}}$. If $p_\mathrm{c}^{(1)},p_\mathrm{c}^{(2)}>p_{\mathrm{ev}}$, then the aqueous humor flows across the trabecular meshwork after the first and second interventions (Fig. 3). In this case, the following system of equations applies:

14 $$\begin{aligned}&p_\mathrm{c}^{(1)}=\frac{\pi D_i^4 p_\mathrm{b}^{(1)}(p_\mathrm{g}-p_{\mathrm{ev}})+128\, L_\mathrm{v} p_\mathrm{g} Q_0 \mu }{\pi D_i^4(p_\mathrm{g}-p_{\mathrm{ev}})+128\, L_\mathrm{v} Q_0 \mu }, \end{aligned}$$

15 $$\begin{aligned}&Q_i^{(1)}=\frac{\pi D_i^4 Q_0 (p_\mathrm{g}-p_\mathrm{b}^{(1)})}{\pi D_i^4 (p_\mathrm{g}-p_{\mathrm{ev}})+128\, L_\mathrm{v} Q_0 \mu }, \end{aligned}$$

16 $$\begin{aligned}&p_\mathrm{c}^{(2)}=\frac{\pi D_\mathrm{h}^4 p_\mathrm{b}^{(2)}(p_\mathrm{g}-p_{\mathrm{ev}})+128\, {{\mathscr {S}}}_2 L_\mathrm{v} p_\mathrm{g} Q_0 \mu }{\pi D_\mathrm{h}^4(p_\mathrm{g}-p_{\mathrm{ev}})+128\, {{\mathscr {S}}}_2 L_\mathrm{v} Q_0 \mu }, \end{aligned}$$

17 $$\begin{aligned}&Q_i^{(2)}=\frac{\pi D_\mathrm{h}^4 Q_0 (p_\mathrm{g}-p_\mathrm{b}^{(2)})}{\pi D_\mathrm{h}^4 (p_\mathrm{g}-p_{\mathrm{ev}})+128\, {{\mathscr {S}}}_2 L_\mathrm{v} Q_0 \mu }, \end{aligned}$$

18 $$\begin{aligned}&\frac{p_\mathrm{b}^{(1)}-p_\mathrm{r}}{Q_i^{(1)}}=\frac{p_\mathrm{b}^{(2)}-p_\mathrm{r}}{Q_i^{(2)}}. \end{aligned}$$

The (known) values of the parameters $\{D_i$,$L_\mathrm{v}$,$Q_0$,$\mu$,$p_{\mathrm{ev}}$,$p_\mathrm{g}$} are the same for the two interventions. The IOP $p_\mathrm{c}^{(1)}$ following the first intervention is known too. In this analysis, one essentially makes use of this pressure to calculate the hydraulic resistance across the belb wall. In fact, that resistance increases with $p_\mathrm{c}^{(1)}$. Notice that Eqs. (16) and (17) differ from their counterparts (14) and (15) in the factor ${{\mathscr {S}}}_2$ and in the fact that $D_i$ has been replaced with $D_\mathrm{h}=D_i-D_\mathrm{s}$.

The system of Eqs. (14)–(18) can be solved to calculate the unknowns $\{Q_i^{(1)}$, $Q_i^{(2)}$, $p_\mathrm{b}^{(1)}$, $p_\mathrm{b}^{(2)}$, ${{\mathscr {S}}}_2\}$. The solution for the shape factor is

19 $$\begin{aligned} {{\mathscr {S}}}_2\left( 1-\frac{D_\mathrm{s}}{D_i}\right) ^{-4}=1+\frac{\pi D_i^4}{128 L_v \mu Q_0} (p_\mathrm{c}^{(2)}-p_\mathrm{c}^{(1)})\frac{(p_\mathrm{g}-p_\mathrm{r})(p_\mathrm{g}-p_{\mathrm{ev}})}{(p_\mathrm{g}-p_c^{(1)})(p_\mathrm{g}-p_c^{(2)})}. \end{aligned}$$

It must be noted that all the parameters on the right side of Eq. (19) are measured in the interventions or can be obtained from the literature. Once calculated the value of ${{\mathscr {S}}}_2 (1-D_\mathrm{s}/D_i)^{-4}$ from Eq. (19), the suture diameter can be inferred from Fig. 2. The value obtained with this procedure must be considered as an upper bound of the suture diameter because we are assuming the conditions leading to the minimum hydraulic resistance. There are several factors that can cause an extra loss of pressure through the implant-bleb system, such as cicatrization of the subconjuntival tissue between the first and second interventions, and bending of the suture inside the implant. The pressure $p_\mathrm{c}^{(2)}$ can be obtained from Eq. (19):

20 $$\begin{aligned} p_\mathrm{c}^{(2)}=\frac{\pi D_i^4 D_\mathrm{h}^4 p_\mathrm{c}^{(1)}(p_\mathrm{g}-p_{\mathrm{ev}})(p_\mathrm{g}-p_\mathrm{r})+128 L_\mathrm{v} \mu Q_0 p_\mathrm{g} (p_\mathrm{c}^{(1)}-p_\mathrm{g})(D_\mathrm{h}^4-D_i^4 {{\mathscr {S}}}_2)}{\pi D_i^4 D_h^4 (p_\mathrm{g}-p_{\mathrm{ev}})(p_\mathrm{g}-p_\mathrm{r})+128 L_\mathrm{v} \mu Q_0 (p_\mathrm{c}^{(1)}-p_\mathrm{g})(D_\mathrm{h}^4-D_i^4 {{\mathscr {S}}}_2)}. \end{aligned}$$

If $p_\mathrm{g}\gg p_c^{(1)}$ and $p_c^{(2)}$, then Eq. (19) reduces to

21 $$\begin{aligned} {{\mathscr {S}}}_2\left( 1-\frac{D_\mathrm{s}}{D_i}\right) ^{-4}=1+\frac{\pi D_i^4}{128 L_v \mu Q_0}(p_\mathrm{c}^{(2)}-p_\mathrm{c}^{(1)}). \end{aligned}$$

The major advantage of this approximation is the fact that it does not require the knowledge of the parameters $p_\mathrm{r}$ and $p_{\mathrm{ev}}$, which are not measured in the clinical procedure and must be taken from the literature. Equation (21) can also be obtained by assuming that the resistance offered by the implant-bleb system is much smaller than that due to the trabecular meshwork and Schlemm’s canal combined, and, therefore, the aqueous humor is almost entirely evacuated by the implant.

**Fig. 4:**
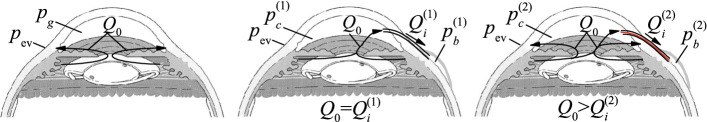
Sketch showing the flow of aqueous humor before surgery (left), after the implanting the drainage device (center), and after introducing a suture into the implant (right). The aqueous humor circulation corresponds to the case in which $p_\mathrm{c}^{(1)}\le p_{\mathrm{ev}}$ and $p_\mathrm{c}^{(2)}>p_{\mathrm{ev}}$, and, therefore, the aqueous humor cannot flow across the trabecular meshwork after the first intervention ($Q_0=Q_i^{(1)}$, $Q_0>Q_i^{(2)}$). The sketch has been adapted from Fig. 1 of Moon et al (2007)

The case $p_c^{(1)}\le p_{\mathrm{ev}}$ *and* $p_\mathrm{c}^{(2)}>p_{\mathrm{ev}}$. If $p_\mathrm{c}^{(1)}\le p_{\mathrm{ev}}$ and $p_\mathrm{c}^{(2)}>p_{\mathrm{ev}}$, then the aqueous humor cannot flow across the trabecular meshwork after the first intervention (Fig. 4). In this case, Eqs. (14) and (15) must be replaced with

22 $$\begin{aligned} p_\mathrm{c}^{(1)}=p_\mathrm{b}^{(1)}+\frac{128\, {{\mathscr {S}}} L_\mathrm{v} \mu Q_0}{\pi D_\mathrm{h}^4} \quad \text {and} \quad Q_i^{(1)}=Q_0, \end{aligned}$$

respectively, and the system of equations analogous to (14)–(18) leads to

23 $$\begin{aligned} {{\mathscr {S}}}_2\left( 1-\frac{D_\mathrm{s}}{D_i}\right) ^{-4}=1+\frac{\pi D_i^4}{128 L_v \mu Q_0}\left[ \frac{p_\mathrm{c}^{(2)}(p_\mathrm{g}-p_{\mathrm{ev}}-p_\mathrm{r})+p_{\mathrm{ev}}p_\mathrm{r}}{p_\mathrm{g}-p_\mathrm{c}^{(2)}}-p_\mathrm{c}^{(1)}\right] . \end{aligned}$$

The pressure $p_\mathrm{c}^{(2)}$ can be obtained from this equation as

24 $$\begin{aligned} p_\mathrm{c}^{(2)}=\frac{\pi D_i^4 D_\mathrm{h}^4 (p_{\mathrm{ev}}p_\mathrm{r}-p_\mathrm{c}^{(1)}p_\mathrm{g})+128 L_\mathrm{v} \mu Q_0 p_\mathrm{g} (D_\mathrm{h}^4-D_i^4 {{\mathscr {S}}}_2)}{\pi D_i^4 D_\mathrm{h}^4 (p_{\mathrm{ev}}+p_\mathrm{r}-p_\mathrm{c}^{(1)}-p_\mathrm{g})+128 L_\mathrm{v} \mu Q_0 (D_\mathrm{h}^4-D_i^4 {{\mathscr {S}}}_2)}. \end{aligned}$$

**Fig. 5:**
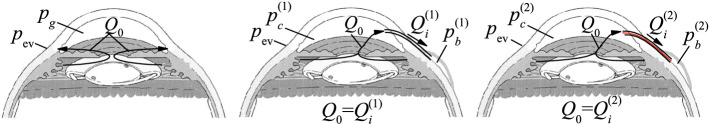
Sketch showing the flow of aqueous humor before surgery (left), after the implanting the drainage device (center), and after introducing a suture into the implant (right). The aqueous humor circulation corresponds to the case in which $p_\mathrm{c}^{(1)},p_\mathrm{c}^{(2)}\le p_{\mathrm{ev}}$, and, therefore, the aqueous humor cannot flow across the trabecular meshwork only after the first and second interventions ($Q_0=Q_i^{(1)}$, $Q_0=Q_i^{(2)}$). The sketch has been adapted from Fig. 1 of Moon et al (2007)

The case $p_\mathrm{c}^{(1)},p_\mathrm{c}^{(2)}\le p_{\mathrm{ev}}$. If $p_\mathrm{c}^{(1)},p_\mathrm{c}^{(2)}\le p_{\mathrm{ev}}$, then the aqueous humor cannot flow across the trabecular meshwork after the first and second interventions (Fig. 5). In this case, the simplification (22) applies to the first intervention. In addition, the counterpart of this simplification for the second intervention also holds:

25 $$\begin{aligned} p_\mathrm{c}^{(2)}=p_\mathrm{b}^{(2)}+\frac{128\, {{\mathscr {S}}} L_\mathrm{v} \mu Q_0}{\pi D_\mathrm{h}^4} \quad \text {and} \quad Q_i^{(2)}=Q_0. \end{aligned}$$

The system of equations analogous to (14)–(18) leads to

26 $$\begin{aligned} {{\mathscr {S}}}_2\left( 1-\frac{D_\mathrm{s}}{D_i}\right) ^{-4}=1+\frac{\pi D_i^4}{128 L_\mathrm{v} \mu Q_0}(p_\mathrm{c}^{(2)}-p_\mathrm{c}^{(1)}), \end{aligned}$$

which coincides with Eq. (21), i.e., the case in which the trabecular meshwork is practically plugged. The pressure $p_\mathrm{c}^{(2)}$ is calculated from Eq. (26) as

27 $$\begin{aligned} p_\mathrm{c}^{(2)}=p_\mathrm{c}^{(1)}+\frac{128 L_\mathrm{v} \mu Q_0 (D_i^4 {{\mathscr {S}}}_2-D_\mathrm{h}^4)}{\pi D_i^4 D_\mathrm{h}^4}. \end{aligned}$$

As can be observed, the use of Eqs. (26) and (27) does not require the knowledge of the parameters $p_\mathrm{r}$ and $p_{\mathrm{ev}}$, which must be estimated from the literature. Interestingly, the case $p_\mathrm{c}^{(1)},p_\mathrm{c}^{(2)}\le p_{\mathrm{ev}}$ is the most frequent one in the clinical practice. The simplicity of this case confers robustness on our analysis.

## Results and discussion

All the results presented in this section were calculated for the aqueous humor viscosity $\mu =0.75$ mPa$\cdot$s, the episcleral venous pressure $p_{\mathrm{ev}}=10.5$ mm Hg, the bleb reference pressure $p_\mathrm{r}=0$, the flow rate $Q_0=1.7$ μl/min evacuated by the trabecular meshwork before the surgery (the flow rate injected by the ciliary body, $Q_{\mathrm{cb}}=2$ μl/min, minus that removed by the uveoscleral pathway, $Q_{\mathrm{up}}=0.15 Q_{\mathrm{cb}}$), and the implant length $L_v=11$ mm. We considered two implant diameters: $D_i=100$ and 305 μm. The values $L_\mathrm{v}=11$ mm and $D_i=305$ μm correspond to a Baerveldt implant, commonly used in the clinical practice, while $D_i=100$ μm has been selected to show the effect of a significant reduction of the implant diameter. We present results for the intermediate value $p_\mathrm{g}=35$ mm Hg of the IOP before surgery. We verified that the conclusions of our analysis are the same for other values of $p_\mathrm{g}$. The results were obtained both when the suture touches the implant tube and when is placed coaxially with it. For the sake of completeness, we also considered values of IOP larger than those corresponding to hypotony.

**Fig. 6:**
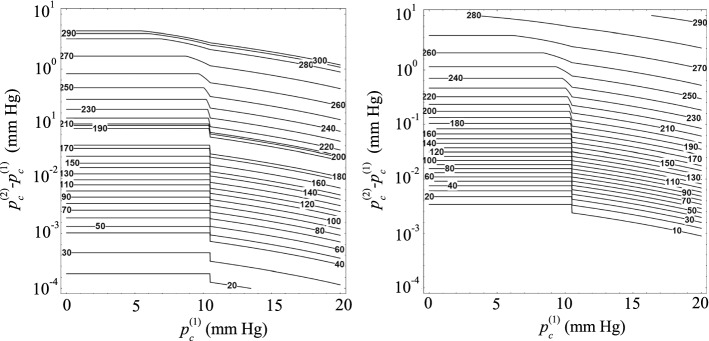
Increase in the IOP after introducing the suture into the implant, $p_\mathrm{c}^{(2)}-p_\mathrm{c}^{(1)}$, as a function of the IOP following the surgery, $p_\mathrm{c}^{(1)}$, for different values of the suture diameter $D_\mathrm{s}$. The results were calculated for $D_i=305$ μm. The left-hand and right-hand contours are the results obtained when the suture touches the wall implant and is placed coaxially with the implant, respectively. The labels indicate the value of $D_\mathrm{s}$ in μm

Figure 6 shows the increase in the IOP after introducing a suture into a Baerveldt implant with $D_i=305$ μm. For small values of $p_\mathrm{c}^{(1)}$, the difference $p_\mathrm{c}^{(2)}-p_\mathrm{c}^{(1)}$ takes a constant value given by Eq. (27). For $p_c^{(1)}>p_{\mathrm{ev}}$, the difference $p_\mathrm{c}^{(2)}-p_\mathrm{c}^{(1)}$ calculated from Eq. (20) decreases as $p_\mathrm{c}^{(1)}$ increases. In fact, the hydraulic resistance offered by flitration bleb increases with $p_\mathrm{c}^{(1)}$, and thus the flow rate evacuated by the implant decreases. This implies that the introduction of the suture has a smaller effect on the IOP as $p_\mathrm{c}^{(1)}$ increases. The interval of $p_\mathrm{c}^{(1)}$ between the two above-mentioned regimes corresponds to the intermediate case $p_\mathrm{c}^{(1)}\le p_{\mathrm{ev}}$ and $p_\mathrm{c}^{(2)}>p_{\mathrm{ev}}$ characterized by Eq. (24). As the suture diameters decreases, the value of $p_\mathrm{c}^{(2)}$ approaches that of $p_\mathrm{c}^{(1)}$, and, therefore, the interval of $p_\mathrm{c}^{(1)}$ corresponding to the intermediate case shrinks around $p_{\mathrm{ev}}$. As can be observed, very small variations of the IOP are obtained for suture diameters $D_\mathrm{s}\lesssim 250$ μm. The IOP increases up to values of the order of several mm Hg only for suture diameters $D_\mathrm{s}\gtrsim 270$ μm. The increase in IOP is significantly greater when the suture is placed coaxially with the implant tube because the hydraulic resistance is larger in this case. Figure 6 may constitute a useful guide for the clinical practice when a straight suture is introduced into a Baerveldt implant because it allows one to estimate the adequate suture diameter as a fuction of both the IOP after surgery and the target value.

**Fig. 7:**
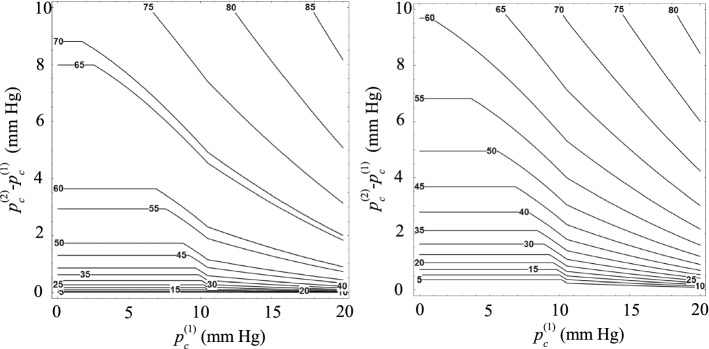
Increase in the IOP after introducing the suture into the implant, $p_\mathrm{c}^{(2)}-p_\mathrm{c}^{(1)}$, as a function of the IOP following the surgery, $p_\mathrm{c}^{(1)}$, for different values of the suture diameter $D_\mathrm{s}$. The results were calculated for $D_i=100$ μm. The left-hand and right-hand graphs are the results obtained when the suture touches the implant tube and is placed coaxially with it, respectively. The labels indicate the value of $D_s$ in μm

The results displayed in Fig. 6 indicate that the IOP after surgery is essentially the same as that built up in the filtering bleb and can hardly be controlled unless we introduce a suture with a diameter slightly smaller than that of the implant. On the contrary, when the implant diameter is reduced down to $D_i=100$ μm (Fig. 7), significant variations of the IOP are obtained by introducing a thin suture into the implant tube. For instance, an increase of 2 mm Hg can be obtained with a suture around 35 μm in diameter placed coaxially with the tube device. In addition, $p_\mathrm{c}^{(2)}$ is fairly sensitive to the suture diameter $D_s$ even for small values of this parameter.

**Fig. 8:**
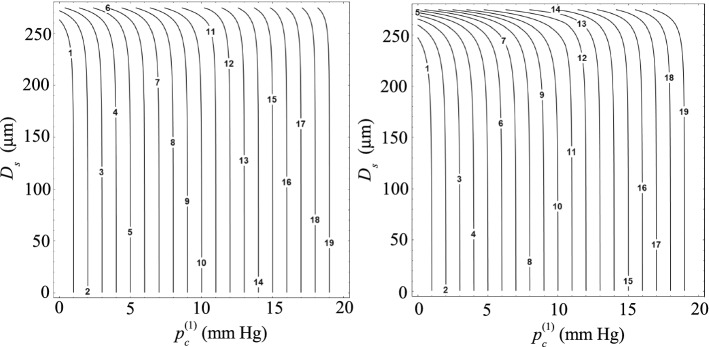
Isocontours of the IOP after introducing the suture into the implant, $p_\mathrm{c}^{(2)}$, as a function of the IOP following the surgery, $p_\mathrm{c}^{(1)}$, and the the suture diameter $D_\mathrm{s}$. The results were calculated for $D_i=305$ μm. The left-hand and right-hand graphs are the results obtained when the suture touches the implant tube and is placed coaxially with it, respectively. The labels indicate the value of $p_\mathrm{c}^{(2)}$ in mm Hg

**Fig. 9:**
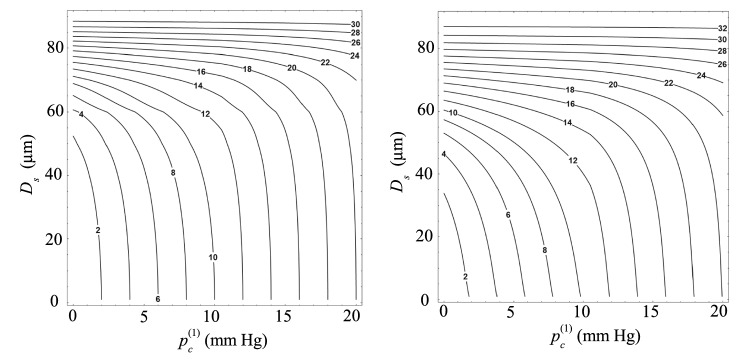
Isocontours of the IOP after introducing the suture into the implant, $p_\mathrm{c}^{(2)}$, as a function of the IOP following the surgery, $p_\mathrm{c}^{(1)}$, and the suture diameter $D_\mathrm{s}$. The results were calculated for $D_i=100$ μm. The left-hand and right-hand graphs are the results obtained when the suture touches the implant tube and is placed coaxially with it, respectively. The labels indicate the value of $p_\mathrm{c}^{(2)}$ in mm Hg

Figures 8 and 9 allow one to predict the increase in the IOP after inserting the suture into the implant as a function of the suture diameter. The conclusions derived from these results are the same as those described above. For $D_i=305$ μm, $p_\mathrm{c}^{(2)}$ is practically the same as $p_\mathrm{c}^{(1)}$ unless the suture diameter takes values close to $D_i$. The effect of the suture diameter is much more noticeable for $D_i=100$ μm.

It must be noted that the IOP approximately equals the pressure in the filtering bleb when a Baerveldt device with $D_i=305$ μm is implanted. In fact, even if one assumes that all the aqueous humor segregated by the ciliary body is drained by the implant, the drop of pressure across that device is smaller than 0.01 mm Hg. This explains why the IOP is hardly affected by the presence of a suture in the device tube unless the suture diameter takes values slightly smaller than 305 μm. When this occurs, small relative variations of $D_\mathrm{s}$ cause large relative variations of the hydraulic characteristic length $D_i-D_\mathrm{s}$, and $p_\mathrm{c}^{(2)}$ becomes very sensitive to $D_\mathrm{s}$.

**Fig. 10:**
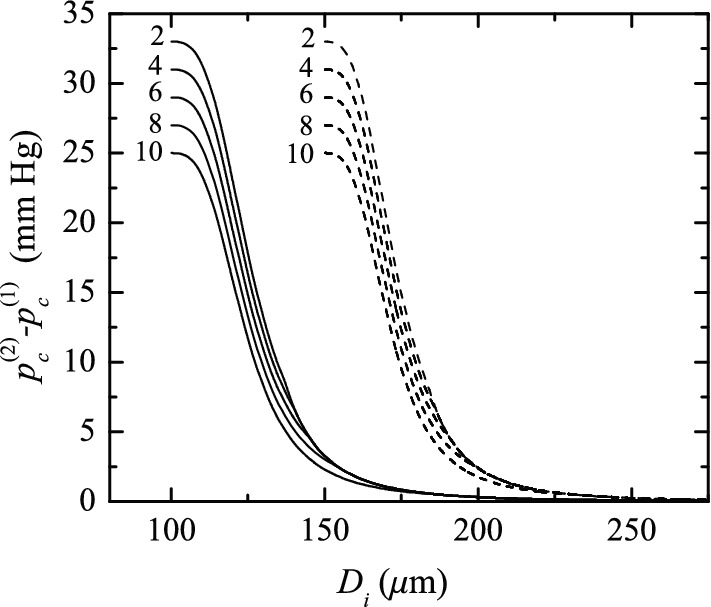
Increment in the IOP after introducing the suture into the implant, $p_\mathrm{c}^{(2)}-p_\mathrm{c}^{(1)}$, as a function of the implant diameter $D_i$. The results were calculated for $D_\mathrm{s}=100$ μm (solid lines) and 150 μm (dashed lines) when the suture is placed coaxially with the implant tube. The labels indicate the value of $p_\mathrm{c}^{(1)}$ in mm Hg

Our theoretical predictions show that negligible increments of the IOP can be obtained when one introduces a suture 100–150 μm in diameter in a Baerveldt device with $D_i=305$ μm (Fig. 10), which differs from some results observed in the clinical practice. We speculate that this may be caused by the bending of the suture inside the tube, which would considerably increase the device’s hydraulic resistance. The fact that the resulting IOP seems to be unpredictable and hardly reproducible under similar conditions (Rietveld and van-der Veen 2004) suggests this possibility.

## Conclusions

In treating glaucoma, the IOP can be reduced by implanting a device to drain the aqueous humor from the anterior chamber to a filtration bleb, where the aqueous humor is absorbed. This intervention produces hypotony when the pressure built up in the filtration bleb takes small values. To correct this, surgeons usually introduce a suture into the device tube, which increases the hydraulic resistance of the device and, therefore, the IOP. This paper has proposed an analytical model to predict the IOP after introducing a straight suture in the glaucoma device. This model may help surgeons in making decisions during hypotony treatment. To illustrate the model’s capabilities, we have examined the increase in the IOP when a suture is introduced in a Baerveldt implant 305 μm in diameter. The results show that the IOP is essentially the same as that built up in the filtering bleb, and negligible increments are obtained by introducing a straight suture unless the suture diameter is slightly smaller than that of the implant. On the contrary, the IOP can be significantly reduced by introducing a thin suture into implants with diameters, say, around 100 μm. In clinical practice, significant but hardly reproducible variations of the IOP are observed when introducing a thin suture into the tube. We hypothesize that this may be due to the bending of the suture inside the implant, which can considerably increase the hydraulic resistance in the device.

The analytical expressions derived in this paper may constitute a useful guide in clinical practice to estimate the appropriate suture diameter as a function of the IOP after surgery and the target value. The most frequent case is that in which the IOP after the two interventions is smaller than the episcleral venous pressure $p_{\mathrm{ev}}=10.5$ mm Hg. This case is especially simple because the aqueous humor cannot flow across the trabecular meshwork and only involves parameter values measured in the interventions or well established in the literature. Our analysis can be easily extended to valved implants provided that the hydraulic resistance offered by the valve is properly characterized.

## Fluid transport and removal in the sub-conjunctival tissue

The flow across the sub-conjunctival tissue of the filtering bleb can be described using Darcy’s law $\mathbf{v}=-K\varvec{\nabla } p_i$, where $\mathbf{v}$ is is homogenized fluid flux per unit area (Darcy’s velocity), *K* is the hydraulic conductivity of the tissue (the ratio of the intrinsic conductivity to the liquid viscosity), and $p_i$ is the hydrostatic pressure in the tissue interstitium. The mass conservation leads to

28 $$\begin{aligned} -\varvec{\nabla }\cdot (K\varvec{\nabla } p_i)=S, \end{aligned}$$

where

29 $$\begin{aligned} S=L_\mathrm{p} s_\mathrm{A} [p_\mathrm{v}-p_i-\sigma (\pi _v-\pi _i)] \end{aligned}$$

represents the volume of fluid produced/removed per unit time and tissue volume by capillaries, $L_\mathrm{p}$ is the hydraulic permeability of the vessel wall, $s_\mathrm{A}$ is the surface area of blood vessel walls per volume of tissue, $p_\mathrm{v}$ is the microvasculature pressure, $\pi _v$ and $\pi _i$ are the microvasculature and interstitium oncotic pressure, respectively, and $\sigma$ is the reflection coefficient. Equation (28) must be integrated in the sub-conjunctival tissue with the boundary conditions

30 $$\begin{aligned}&p_i=p_\mathrm{b} \quad \text {on the internal bundary}, \end{aligned}$$

31 $$\begin{aligned}&\frac{\partial p_i}{\partial n}=0 \quad \text {on the external boundary}, \end{aligned}$$

where *n* represents the direction normal to the boundary surface.

Let us now define the dimensionless pressure $\varPi _i\equiv (p_i-p_\mathrm{r})/(p_\mathrm{b}-p_\mathrm{r}),$ where $p_\mathrm{r}\equiv p_v-\sigma (\pi _v-\pi _i)$ is a constant value. The dimensionless pressure verifies the set of equations

32a $$\begin{aligned}&\varvec{\nabla }\cdot (K\varvec{\nabla } \varPi _i)=L_\mathrm{p} s_\mathrm{A} \varPi _i, \end{aligned}$$

32b $$\begin{aligned}&\varPi _i=1 \quad \text {on the internal bundary} \end{aligned}$$

32c $$\begin{aligned}&\frac{\partial \varPi _i}{\partial n}=0 \quad \text {on the external boundary} \end{aligned}$$

For a given sub-conjunctival tissue, the solution $\varPi _i(\mathbf{r})$ of (32) is unique. Under steady conditions, the flow rate absorbed by the sub-conjunctival tissue equals that injected into the bleb by the implant. Therefore,

33 $$\begin{aligned} Q_i=-\int _V S\, \mathrm{d}V=(p_b-p_r)\int _V L_\mathrm{p} s_\mathrm{A} \varPi _i\, \mathrm{d}V=(p_\mathrm{b}-p_\mathrm{r})/R_\mathrm{b}, \end{aligned}$$

where *V* is the volume of the sub-conjunctival tissue, and $R_\mathrm{b}=(\int _V L_\mathrm{p} s_\mathrm{A} \varPi _i\, \mathrm{d}V)^{-1}$ is its effective hydraulic resistance.

The filtering bleb following implantation surgery of a glaucoma drainage device may exhibit a complex morphology depending on both the implanted device and the patient (Iwasaki et al 2017). However, and as shown in Sect. 3, neither the morphology nor the hydraulic properties of the bleb intervene in our study. In fact, the only requisite demanded by our analysis is that both the effective hydraulic resistance $R_\mathrm{v}$ and the reference pressure $p_\mathrm{r}$ remain constant after the implantation surgery.
